# Navigating the Nose-to-Brain Route: A Systematic Review on Lipid-Based Nanocarriers for Central Nervous System Disorders

**DOI:** 10.3390/pharmaceutics16030329

**Published:** 2024-02-27

**Authors:** Edoardo Agosti, Marco Zeppieri, Sara Antonietti, Luigi Battaglia, Tamara Ius, Caterina Gagliano, Marco Maria Fontanella, Pier Paolo Panciani

**Affiliations:** 1 Division of Neurosurgery, Department of Medical and Surgical Specialties, Radiological Sciences and Public Health, University of Brescia, Piazza Spedali Civili 1, 25123 Brescia, Italy; edoardo_agosti@libero.it (E.A.);; 2 Department of Ophthalmology, University Hospital of Udine, p.le S. Maria della Misericordia 15, 33100 Udine, Italy; 3 Department of Drug Science and Technology, University of Torino, 10124 Torino, Italy; 4 Neurosurgery Unit, Head-Neck and Neuroscience Department University Hospital of Udine, p.le S. Maria della Misericordia 15, 33100 Udine, Italy; 5 Faculty of Medicine and Surgery, University of Enna “Kore”, Piazza dell’Università, 94100 Enna, Italy; 6 Eye Clinic, Catania University San Marco Hospital, Viale Carlo Azeglio Ciampi, 95121 Catania, Italy

**Keywords:** neurodegenerative disorders, neuro-oncological diseases, blood–brain barrier, lipid nanoparticles, intranasal administration, clinical outcomes, systematic reviews

## Abstract

Background: The blood–brain barrier (BBB) regulates brain substance entry, posing challenges for treating brain diseases. Traditional methods face limitations, leading to the exploration of non-invasive intranasal drug delivery. This approach exploits the direct nose-to-brain connection, overcoming BBB restrictions. Intranasal delivery enhances drug bioavailability, reduces dosage, and minimizes systemic side effects. Notably, lipid nanoparticles, such as solid lipid nanoparticles and nanostructured lipid carriers, offer advantages like improved stability and controlled release. Their nanoscale size facilitates efficient drug loading, enhancing solubility and bioavailability. Tailored lipid compositions enable optimal drug release, which is crucial for chronic brain diseases. This review assesses lipid nanoparticles in treating neuro-oncological and neurodegenerative conditions, providing insights for effective nose-to-brain drug delivery. Methods: A systematic search was conducted across major medical databases (PubMed, Ovid MEDLINE, and Scopus) up to 6 January 2024. The search strategy utilized relevant Medical Subject Heading (MeSH) terms and keywords related to “lipid nanoparticles”, “intranasal administration”, “neuro-oncological diseases”, and “neurodegenerative disorders”. This review consists of studies in vitro, in vivo, or ex vivo on the intranasal administration of lipid-based nanocarriers for the treatment of brain diseases. Results: Out of the initial 891 papers identified, 26 articles met the eligibility criteria after a rigorous analysis. The exclusion of 360 articles was due to reasons such as irrelevance, non-reporting selected outcomes, the article being a systematic literature review or meta-analysis, and lack of method/results details. This systematic literature review, focusing on nose-to-brain drug delivery via lipid-based nanocarriers for neuro-oncological, neurodegenerative, and other brain diseases, encompassed 60 studies. A temporal distribution analysis indicated a peak in research interest between 2018 and 2020 (28.3%), with a steady increase over time. Regarding drug categories, Alzheimer’s disease was prominent (26.7%), followed by antiblastic drugs (25.0%). Among the 65 drugs investigated, Rivastigmine, Doxorubicin, and Carmustine were the most studied (5.0%), showcasing a diverse approach to neurological disorders. Notably, solid lipid nanoparticles (SLNs) were predominant (65.0%), followed by nanostructured lipid carriers (NLCs) (28.3%), highlighting their efficacy in intranasal drug delivery. Various lipids were employed, with glyceryl monostearate being prominent (20.0%), indicating preferences in formulation. Performance assessment assays were balanced, with in vivo studies taking precedence (43.3%), emphasizing the translation of findings to complex biological systems for potential clinical applications. Conclusions: This systematic review reveals the transformative potential of intranasal lipid nanoparticles in treating brain diseases, overcoming the BBB. Positive outcomes highlight the effectiveness of SLNs and NLCs, which are promising new approaches for ailments from AD to stroke and gliomas. While celebrating progress, addressing challenges like nanoparticle toxicity is also crucial.

## 1. Introduction

The blood–brain barrier (BBB) is a vital physiological barrier that regulates the entry of substances into the brain. However, this protective mechanism also poses a significant challenge for the effective treatment of brain diseases, including neuro-oncological conditions and neurodegenerative disorders. Indeed, the intricate structure of the BBB restricts the passage of therapeutic agents, limiting the efficacy of pharmacological interventions [1]. In the quest to overcome these challenges, alternative strategies to traditional drug delivery methods have been explored. Invasive techniques such as transcranial drug delivery and disruption of the BBB have been investigated, but they often face limited success and numerous hurdles, including safety concerns and the potential for adverse effects [2]. In this context, intranasal drug delivery emerges as a promising non-invasive approach that offers direct access to the brain, bypassing the systemic circulation.

Intranasal drug delivery provides a non-invasive route that exploits the direct connection between the nasal cavity and the central nervous system (CNS). This method offers a unique advantage by circumventing the BBB, allowing drugs to reach the brain more effectively [3]. The nasal route provides a direct pathway for drug delivery to the CNS through both intra- and extraneuronal pathways. This direct access enhances drug bioavailability, reducing the required dosage and minimizing systemic side effects [4]. One of the key advantages of intranasal drug delivery is its ability to deliver therapeutic agents specifically to the brain, while avoiding other organs, thus enhancing drug targeting and minimizing off-target effects [5]. Additionally, this approach allows for a rapid onset of action, making it particularly attractive for the treatment of acute conditions.

Among the various drug delivery systems explored for intranasal administration, lipid nanoparticles have garnered significant attention due to their unique properties and advantages. Lipid nanoparticles, including solid lipid nanoparticles (SLNs) and nanostructured lipid carriers (NLCs), have been proven to be efficient drug carriers, with enhanced stability, controlled release, and improved drug penetration [6]. The reduced invasiveness of intranasal drug delivery, combined with the favorable characteristics of lipid nanoparticles, addresses critical challenges in conventional brain drug delivery. Lipid nanoparticles offer enhanced biocompatibility, reducing the risk of adverse reactions and improving patient tolerance [7].

Lipid nanoparticles exhibit several advantages for nose-to-brain drug delivery. Their nanoscale size allows for efficient drug loading and encapsulation, facilitating enhanced drug solubility and bioavailability [8]. Moreover, lipid nanoparticles can protect sensitive drugs from degradation, ensuring their stability during transport to the brain [9]. Moreover, the lipid composition of these nanoparticles can be tailored to achieve optimal drug release profiles, providing sustained therapeutic levels over an extended period. This feature is particularly crucial for the treatment of chronic brain diseases, including neurodegenerative disorders [10].

This systematic literature review aims to comprehensively evaluate the current state of treatment options based on lipid nanoparticles for major brain diseases, with a focus on neuro-oncological and neurodegenerative conditions. The review explores the fundamental preparation methods, challenges encountered, and evaluation techniques for lipid-based drug delivery systems. By consolidating the existing knowledge, we aim to unveil the techniques and pathways involved in nose-to-brain drug delivery using lipid nanoparticles and provide preliminary insights for the effective treatment of various brain pathologies.

## 2. Materials and Methods

### 2.1. Literature Review

The systematic review was performed following the Preferred Reporting Items for Systematic Reviews and Meta-Analysis (PRISMA) guidelines [11]. Two authors (E.A. and S.A.) performed a systematically comprehensive literature search of the databases PubMed, Ovid MEDLINE, and Scopus. The first literature search was performed on 15 December 2023, and the search was updated on 6 January 2024. A combination of keyword searches was performed to generate a search strategy. The search keywords, including “lipid nanoparticles”, “intranasal administration”, “neuro-oncological diseases”, and “neurodegenerative disorders”, were used in both AND and OR combinations. Studies were retrieved using the following Medical Subject Heading (MeSH) terms and Boolean operators: (lipid nanoparticles OR solid lipid nanoparticles OR nanostructured lipid carriers) AND (neuro-oncological disease OR glioma OR neurodegenerative disorder OR brain disease) AND (nose-to-brain OR intranasal administration). Other pertinent articles were identified through reference analysis of selected papers. All studies were selected based on the following inclusion criteria: (1) English language; (2) studies in vitro, in vivo, or ex vivo on the intranasal administration of lipid-based nanocarriers for the treatment of brain diseases; and (3) studies including details on drug, lipid-based nanocarrier, and biodistribution modality. The following exclusion criteria were employed: (1) editorials, case reports, case series, cohort studies, literature reviews, and meta-analyses; and (2) studies that do not clearly define the methods and/or results.

The list of identified studies was imported into Endnote X9, and duplicates were removed. Two independent researchers (E.A. and S.A.) checked the results according to the inclusion and exclusion criteria. A third reviewer (P.P.P.) resolved all disagreements. Then, eligible articles were subject to full-text screening.

### 2.2. Data Extraction

For each study, we abstracted the following information: authors, year of publication, drug category, lipid-based nanocarriers type, and assays performed.

### 2.3. Outcomes

The primary outcomes were the definition of the main categories of lipid-based nanocarriers utilized for drug transport to the CNS and the testing methodologies employed to assess them. Secondary outcomes were the type of drug delivered and the brain diseases treated.

### 2.4. Risk-of-Bias Assessment

**Figure 1:**
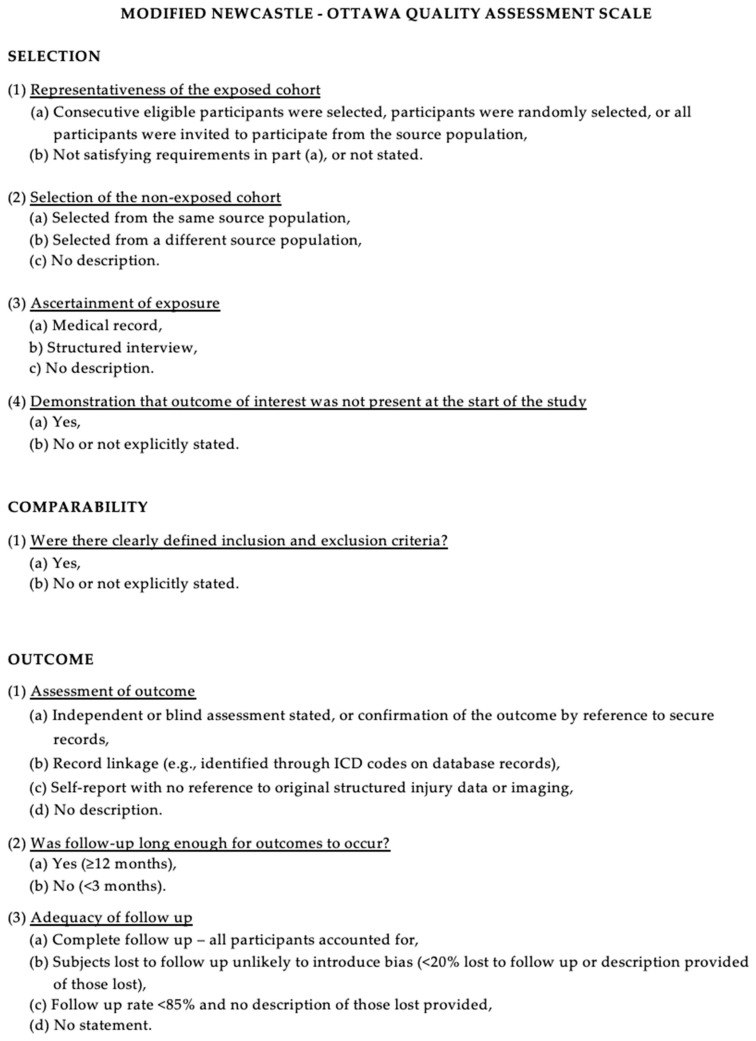
The Modified NOS.

The Newcastle–Ottawa Scale (NOS) [12] was used to assess the quality of the included studies. Quality assessment was performed by assessing the selection criteria, comparability of the study, and outcome assessment. The ideal score was 9. Higher scores indicated better quality of studies. Studies receiving 7 or more points were considered high-quality studies. Two authors (E.A. and P.P.P.) performed the quality assessment independently. When discrepancies arose, papers were re-examined by the third author (Figure 1).

### 2.5. Statistical Analysis

Descriptive statistics were reported, including ranges and percentages. All statistical analyses were performed using the R statistical package v3.4.1 (http://www.r-project.org (accessed on 20 February 2024)).

## 3. Results

### 3.1. Literature Review

**Figure 2:**
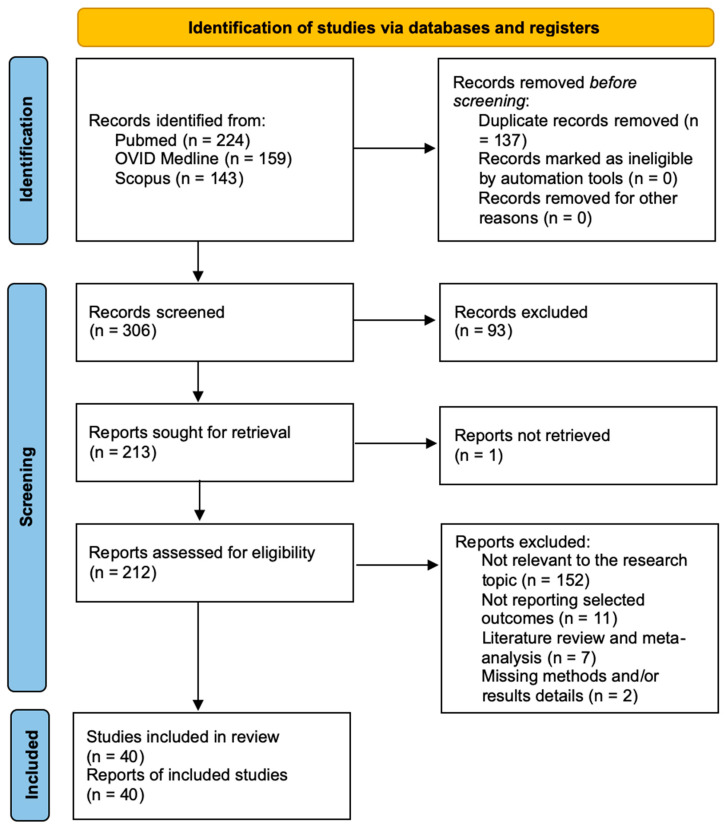
PRISMA flowchart.

A total of 891 papers were identified after duplicate removal. After title and abstract analysis, 388 articles were identified for full-text analysis. Eligibility was assessed for 386 articles and ascertained for 26 articles. The remaining 360 articles were excluded for the following reasons: (1) not relevant to the research topic (345 articles), (2) articles non-reporting selected outcomes (9 articles), (3) systematic literature review or meta-analysis (5 articles), and (4) lack of method and/or results details (1 article). All studies included in the analysis had at least one or more outcome measures available for one or more of the patient groups analyzed. Figure 2 shows the flowchart according to the PRISMA statement.

**Figure A1:**
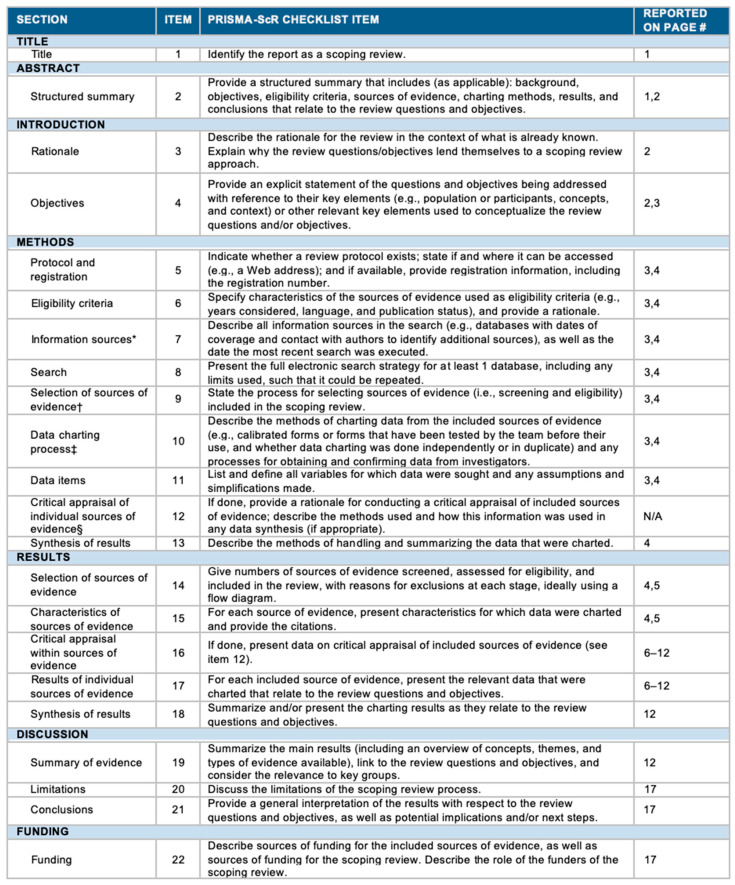
The PRISMA-ScR checklist. * Where sources of evidence (see second footnote) are compiled from, such as bibliographic databases, social media platforms, and Web sites. † A more inclusive/heterogeneous term used to account for the different types of evidence or data sources (e.g., quantitative and/or qualitative research, expert opinion, and policy documents) that may be eligible in a scoping review as opposed to only studies. This is not to be confused with information sources (see first footnote). ‡ The frameworks by Arksey and O’Malley (6) and Levac and colleagues (7) and the JBI guidance (4, 5) refer to the process of data extraction in a scoping review as data charting. § The process of systematically examining research evidence to assess its validity, results, and relevance before using it to inform a decision. This term is used for items 12 and 19 instead of "risk of bias" (which is more applicable to systematic reviews of interventions) to include and acknowledge the various sources of evidence that may be used in a scoping review (e.g., quantitative and/or qualitative research, expert opinion, and policy document).

The PRISMA Extension for Scoping Reviews (PRISMA-ScR) checklist is available in Appendix A, Figure A1.

### 3.2. Data Analysis

A summary of the included studies reporting on the intranasal administration of lipid-based nanocarriers for brain diseases is presented in Table 1, Table 2 and Table 3 for neuro-oncological lesions, neurodegenerative disorders, and other brain diseases, respectively.

This systematic literature review focuses on nose-to-brain drug delivery via lipid-based nanocarriers for the treatment of neuro-oncological, neurodegenerative, and other brain diseases. A meticulous analysis of the data presented in the provided table sheds light on key parameters, including the year of publication, drug category, drug used, lipid nanocarrier type, lipid employed, and assays performed. This review encompasses 60 studies focusing on targeted therapies for craniopharyngiomas. Table 1 specifically addresses neuro-oncological lesions, featuring 10 studies; Table 2 concentrates on papillary neurodegenerative disorders, involving 19 studies; and Table 3 summarizes the data on other brain diseases, accounting for 11 studies.

The studies reviewed span from 2009 to 2023, with a peak observed between 2018 and 2020 (11 studies, 27.5%), indicating a steady increase in research interest over time. The breakdown of publications across time periods is as follows: 2011–2015, 12 studies (30.0%); 2016–2020, 23 studies (57.5%); and 2021–2023, 5 studies (12.5%). This temporal distribution suggests a growing momentum and recent emphasis on exploring intranasal lipid nanocarriers for neurotherapeutics.

The analysis of drug categories across the reviewed studies reveals a diverse landscape, with a predominant focus on Alzheimer’s disease (AD), featured in 11 studies (27.5%). Following closely is the category of antiblastic, comprising nine studies (22.5%), indicating a substantial emphasis on anticancer therapeutics. Antiepileptic drugs are explored in three studies (7.5%), while antipsychotic and antiviral categories each contribute two studies (5.0%). A single study each (2.5%) is dedicated to hepatoprotective, antiviral, antibacterial, antioxidative stress, anti-angiogenic monoclonal antibody, anti-inflammatory, analgesic, anti-ischemic, and nucleic acid drug. Additionally, a combined focus on anti-AD and anti-ALS is observed in two studies (5.0%), showcasing a nuanced approach to neurodegenerative conditions. The distribution underscores the breadth of research interests, spanning neurodegenerative diseases, oncology, antiviral strategies, and diverse therapeutic areas, contributing to the multifaceted exploration of intranasal lipid nanocarriers for targeted drug delivery.

The exploration of drug categories within the reviewed publications reveals a spectrum of specific drugs investigated for intranasal lipid nanocarrier delivery. Rivastigmine, Doxorubicin, and Carmustine emerge as the most frequently studied, each featured in three publications (7.5%). Temozolomide follows closely with two publications (5.0%), while valproic acid, ondansetron, alprazolam, curcumin, vinpocetine, olanzapine, efavirenz, agomelatine, asenapine maleate, ferulic acid, carbamazepine, topiramate, saquinavir mesylate, risperidone, and ketoconazole are each investigated in one publication (2.5%). This rich diversity of drugs underscores the comprehensive approach to addressing various neurological disorders through intranasal lipid nanocarrier delivery. The distribution reflects a nuanced understanding of drug-specific applications, contributing to the intricate landscape of targeted therapies for neurodegenerative, oncological, and other brain diseases.

The studies analyzed various lipid nanocarrier types, revealing a predominant utilization of SLNs, featured in 29 studies (72.5%), and NLC, employed in 11 studies (27.5%). Lipid-based nanosystems, glycol chitosan-coated nanostructured lipid carriers, cubosomes, and liposomes were each explored in 2.5% of the studies. The prevalence of SLN and NLC emerges as noteworthy, indicating their recognized efficacy and suitability for intranasal drug delivery. This distribution underscores the significance of these lipid nanocarriers in advancing the field, reflecting their potential as promising vehicles for targeted drug delivery to the brain.

Across the studies, various lipids were employed in the preparation of lipid nanocarriers, showcasing a diverse landscape of lipid choices. The distribution of lipid types is as follows: glyceryl monostearate featured prominently in 20.0% of the studies (eight publications), while Compritol 888 ATO was utilized in 10.0% of the studies (four publications). Tripalmitin was employed in 7.5% of the investigations (three publications), and cetyl palmitate and stearic acid each constituted 5.0% of the studies (two publications each). Capryol PGMC, D-α-tocopherol polyethylene glycol 1000 succinate, Myristyl Mystriate, mono-oleic acid, behenic acid, soy lecithin with glyceryl behenate, oleic acid, sphingosylphosphorylcholine, and Capmul MCM C8 each represented 2.5% of the studies (one publication each). Notably, glyceryl monostearate, Compritol 888 ATO, and tripalmitin emerged as frequently employed lipids, indicating their popularity and preference in formulating lipid nanocarriers for intranasal delivery. This trend underscores the importance of lipid selection in designing effective carriers for targeted drug delivery to the brain, reflecting considerations of biocompatibility, stability, and delivery efficiency.

In the realm of assessing the performance and efficacy of intranasally administered lipid nanocarriers, a variety of assays were employed across studies. Distinguishing among the assays performed, the categorization encompasses in vitro, ex vivo, and in vivo studies. The distribution of these assays reveals a balanced approach: in vitro studies accounted for 40.0% of the investigations (16 studies), ex vivo studies comprised 25.0% of the total (10 studies), and in vivo studies took precedence in 35.0% of the studies (14 investigations). This nuanced breakdown underscores the comprehensive nature of the research endeavors, with a notable emphasis on in vivo studies. The dominance of in vivo investigations suggests a keen interest in translating findings from controlled laboratory settings to the intricacies of more complex biological systems. This approach is pivotal for bridging the gap between experimental evidence and potential clinical applications, fostering a deeper understanding of the practical implications and therapeutic potential of intranasal lipid nanocarrier delivery systems.

## 4. Discussion

### 4.1. SLNs and NLCs for Nose-to-Brain Drug Delivery

Delivering drugs to the brain presents formidable challenges due to the protective BBB. Recent advancements in nanotechnology have led to promising approaches, with intranasal delivery gaining attention as a non-invasive route. Within this realm, SLNs and NLCs have emerged as potential vehicles. This discussion explores the pathways and feasibilities of SLNs and NLCs for nose-to-brain drug delivery, drawing insights from recent studies [28,30,51].

Intranasal drug delivery provides a direct and non-invasive route to the CNS, circumventing the challenges posed by the BBB. This approach, highlighted by Dhuria et al. [52] and Illum [53], offers a unique advantage by allowing drugs to bypass the systemic circulation [52,53]. Intranasal delivery’s potential is particularly crucial for treating neurological disorders such as Alzheimer’s and Parkinson’s diseases, where precise drug targeting is imperative [51].

SLNs represent a promising avenue for drug delivery to the brain. These nanoparticles, as introduced by Battaglia et al. [54], are composed of lipids that are generally recognized as safe (GRAS), ensuring biocompatibility [54]. Their advantages include ease of functionalization and bioadhesive properties, allowing prolonged drug residence time on the nasal mucosa [55]. Additionally, SLNs offer scalable production and can be steam sterilized, enhancing their industrial applicability [56]. Recent studies have explored the potential of SLNs for nose-to-brain drug delivery. For instance, Chandra Bhatt et al. [28] demonstrated successful nose-to-brain delivery of astaxanthin-loaded SLNs, emphasizing the fabrication, radio-labeling, optimization, and biological studies [28]. Battaglia et al. [51] provided insights into the applications of SLNs for targeted brain drug delivery, particularly in treating neurodegenerative diseases [51]. Rassu et al. [30] showcased the potential of SLNs for Alzheimer’s therapy by delivering BACE1 siRNA directly to the brain [30]. These studies collectively underline the versatility and efficacy of SLNs in diverse therapeutic applications.

SLNs present certain feasibilities and challenges for nose-to-brain drug delivery. The biocompatibility of SLNs, derived from GRAS excipients, ensures minimal irritation and toxicity [55]. The bioadhesive properties of SLNs enhance nasal mucosa interactions, contributing to improved drug absorption [55]. However, challenges persist, such as the limited drug payload and the need for potent drugs to ensure successful administration. Individual variability in nasal uptake and susceptibility to altered nasal mucosa further pose challenges [51]. Nevertheless, the non-invasiveness of intranasal delivery and the efficient BBB bypassing provided by SLNs make them promising candidates for future drug development [51].

Nanostructured lipid carriers represent a further advancement in lipid nanoparticle technology. As highlighted by Müller et al. [57], NLCs are designed to overcome certain limitations of SLNs, particularly related to drug loading capacity [57]. The incorporation of liquid lipids into the lipid matrix of NLCs allows for increased drug loading and improved stability [57]. This innovative design enhances the feasibility of using lipid nanoparticles for nose-to-brain drug delivery. Recent studies have explored the applications of NLCs in nose-to-brain drug delivery. Bhatt et al. [26] developed and delivered rosmarinic acid-loaded NLCs for Huntington’s disease, showcasing both safety and efficacy [26]. Esposito et al. [31] explored nanoformulations for dimethyl fumarate, demonstrating promising physicochemical characterization and in vivo behavior for neurological disorder treatment [31]. Patel et al. [40] focused on the brain targeting of risperidone-loaded NLCs, illustrating the potential of this approach in antipsychotic drug delivery [40]. These studies collectively underscore the versatility and efficacy of NLCs in diverse therapeutic applications.

### 4.2. Intranasal Delivery of Lipid-Based Nanocarriers for Neuro-Oncological Diseases

In recent years, intranasal drug delivery has emerged as a promising avenue for efficiently targeting the CNS, particularly for the treatment of neuro-oncological lesions. The intricate challenges associated with treating brain diseases, compounded by the restrictive BBB, have necessitated innovative drug delivery strategies [15,16,17,21].

Solid lipid nanoparticles have garnered attention for their versatility in drug delivery, particularly to the brain. In the context of neuro-oncology, SLNs present a promising platform. As Battaglia et al. [51] discuss, SLNs offer a better biocompatibility profile, making them suitable for intranasal delivery. This is crucial given the sensitivity of neural tissues and the need to minimize potential irritation or toxicity. The ease of functionalization and bioadhesive properties of SLNs further enhance their suitability for nasal drug delivery [51]. In the realm of neuro-oncological lesions, SLNs have demonstrated efficacy in delivering a range of therapeutic agents. For instance, in the treatment of glioblastoma multiforme (GBM), a highly aggressive brain tumor, SLNs have been explored as carriers for pharmacological agents [58]. The ability of SLNs to encapsulate drugs with diverse properties, coupled with their biocompatibility, positions them as promising candidates for targeted drug delivery to brain tumors.

Nanostructured lipid carriers (NLCs) represent an evolution of SLNs, designed to overcome some of the limitations associated with the latter. The incorporation of both solid and liquid lipids in NLCs results in a more structurally stable system, offering improved drug loading capacities and controlled release [51]. This is particularly relevant in the context of neuro-oncology, where sustained and targeted drug release is essential for therapeutic efficacy. NLCs have demonstrated success in delivering a variety of drugs to the brain. In the treatment of gliomas, which are a type of tumor originating from glial cells, NLCs have shown promise in enhancing drug bioavailability and overcoming the challenges posed by the BBB [13,30]. The ability of NLCs to encapsulate both hydrophobic and hydrophilic drugs widens the therapeutic scope for addressing the heterogeneous nature of neuro-oncological lesions.

### 4.3. Intranasal Delivery of Lipid-Based Nanocarriers for Neurodegenerative Disorders

Neurodegenerative disorders present a formidable barrier to effective drug delivery, primarily due to the protective BBB that restricts the passage of therapeutic agents. However, recent advancements in nanotechnology have kindled interest in innovative administration routes, with a spotlight on the intranasal delivery of lipid-based nanocarriers as a promising strategy [23,24,35,36,37,38,52,53].

Solid lipid nanoparticles have garnered attention for their biocompatibility and facile functionalization. Studies, exemplified by Battaglia et al. [51], underscore the potential of SLNs in intranasal drug delivery for neurodegenerative diseases. SLNs provide an adaptable platform for drugs targeting Alzheimer’s disease (AD), Parkinson’s disease (PD), Huntington’s disease (HD), and amyotrophic lateral sclerosis (ALS) [28,29,30,31,32]. Their ease of functionalization and bioadhesive properties make SLNs ideal for tailoring drug delivery systems [51,59]. Preclinical studies involving the intranasal administration of SLNs loaded with specific drugs, such as Rivastigmine for AD [26], ropinirole for PD [2], and rosmarinic acid for HD [26], have demonstrated promising results. The ability to enhance permeation into the CNS through functionalization addresses the limitations of conventional drug delivery methods [51].

Nanostructured lipid carriers represent an alternative in intranasal drug delivery for neurodegenerative disorders [25,33,34,39]. NLC formulation, involving a blend of solid and liquid lipids, provides advantages in terms of drug loading capacity and controlled release. Studies conducted by Van Woensel et al. [58] and Esposito et al. [31] emphasize the potential of NLCs in overcoming the limitations of traditional drug formulations for CNS disorders. NLCs have been investigated for delivering drugs like dimethyl fumarate for neurodegenerative diseases [31], risperidone for psychiatric disorders, and vinpocetine for anti-ischemic effects [44]. These studies underscore the versatility of NLCs in encapsulating various therapeutic agents and their potential for targeted drug delivery to the brain.

### 4.4. Nose-to-Brain Delivery of SLNs and NLCs for Brain Diseases: In Vitro Studies

In vitro studies serve as the foundational bedrock for unraveling the complexities of SLNs and NLCs in the context of nose-to-brain drug delivery. The work of Patel et al. [60] significantly contributes to understanding the intricacies of SLN-based formulations in neurological drug delivery. In their in vitro experiments, Patel and colleagues shed light on the remarkable ability of SLNs to encapsulate a diverse array of drugs, emphasizing their versatility for neurological applications. The study further highlighted sustained release patterns exhibited by SLNs, showcasing enhanced drug permeation across cell membranes. This in vitro exploration by Patel et al. [60] not only established SLNs as promising carriers for neurological therapeutics but also revealed crucial insights into their controlled drug release mechanisms. Additionally, Lauzon et al. [59] expanded on the application of SLNs, specifically emphasizing their biocompatibility and ease of functionalization. Their in vitro investigations provided further evidence of SLNs’ potential for tailored drug delivery systems, a critical aspect in the pursuit of precision medicine for neurodegenerative disorders.

Shadab et al. [61] delved into the realm of NLCs, focusing on their versatility and improved drug-loading capacities. Through in vitro experiments, the researchers demonstrated the enhanced cellular uptake and sustained drug release characteristics of NLCs. These findings not only underscored the feasibility of NLCs as efficient drug carriers but also highlighted their potential for prolonged therapeutic effects. The controlled release observed in NLCs at the cellular level aligns with the goals of nose-to-brain drug delivery, ensuring sustained drug concentrations within the brain, while minimizing systemic exposure. Furthermore, Gupta et al. [45] explored the systematic formulation and optimization of Efavirenz-loaded SLNs, using high-pressure homogenization. Their in vitro studies were pivotal for understanding the potential of SLNs for brain targeting, emphasizing the significance of formulation strategies in enhancing bioavailability and therapeutic efficacy.

The collective findings from these in vitro studies reaffirm the promise of SLNs and NLCs as effective carriers for nose-to-brain drug delivery. The ability to encapsulate diverse drugs, exhibit sustained release, and enhance cellular uptake positions these lipid nanoparticles as potent candidates for overcoming the challenges posed by the blood–brain barrier.

### 4.5. Nose-to-Brain Delivery of SLNs and NLCs for Brain Diseases: Ex Vivo Studies

The journey of SLNs and NLCs from in vitro promise to potential clinical applications involves a critical phase of ex vivo investigations [14,19,22,25]. Patel et al. [60] extended their exploration of SLNs into the ex vivo domain, aiming to bridge the gap between cellular mechanisms and the complex biological environment of the nasal mucosa. Their experiments elucidated the behavior of SLN-based formulations when exposed to nasal tissue, emphasizing sustained drug release patterns and enhanced permeation. These ex vivo findings are pivotal, offering insights into the translational potential of SLNs for nose-to-brain drug delivery. Moreover, Lauzon et al. [59] contributed to the ex vivo landscape by assessing the biocompatibility and functionalization potential of SLNs. Their work established a connection between the physical characteristics of SLNs and their interaction with nasal tissues. The ex vivo studies highlighted the feasibility of tailoring SLNs for specific drug delivery needs, marking a crucial step towards personalized therapeutic strategies for neurodegenerative disorders.

Shadab et al. [62] expanded their investigation of NLCs into the ex vivo realm, providing valuable insights into how these lipid carriers interact with nasal tissues. The experiments demonstrated enhanced cellular uptake and sustained drug release characteristics, mirroring the in vitro findings. The ex vivo studies reinforce the potential of NLCs to maintain their advantageous features within the complex nasal environment, a crucial aspect for successful nose-to-brain drug delivery. Furthermore, Van Woensel et al. [58] conducted ex vivo experiments assessing the potential of NLCs in overcoming the challenges associated with traditional CNS drug formulations. Their findings provided a bridge between cellular mechanisms and the intact nasal mucosa, emphasizing the applicability of NLCs in navigating the nasal route for efficient drug delivery to the brain.

### 4.6. Nose-to-Brain Delivery of SLNs and NLCs for Brain Diseases: In Vivo Studies

Numerous in vivo studies have contributed compelling evidence supporting the efficacy of SLNs and NLCs in the context of nose-to-brain drug delivery [41,42,43,45,46]. Devkar et al. [62] conducted a groundbreaking investigation focused on engineering nanostructured lipid carriers to facilitate the efficient nose-to-brain delivery of ondansetron HCl. The study introduced Delonix regia gum, a natural mucoadhesive polymer, into the formulation, thereby extending the residence time of the carriers within the nasal cavity. This innovative approach significantly enhanced drug absorption, offering a promising strategy for optimizing therapeutic outcomes. In a separate study, Khan et al. [14] explored the intranasal delivery of temozolomide, a drug with implications for brain disorders, utilizing lipid-based nanoparticles. The researchers conducted comprehensive brain pharmacokinetic and scintigraphic analyses, affirming the targeted delivery and accumulation of temozolomide. This study underscored the potential of lipid-based formulations as an effective strategy for precise central nervous system targeting, showcasing their versatility across diverse therapeutic applications. Adding to the body of evidence, Sivadasu et al. [63] delved into the exploration of ziprasidone hydrochloride-loaded nanostructured lipid carriers for intranasal delivery. The formulation not only demonstrated promising results in terms of optimization but also exhibited notable outcomes in rigorous in vivo studies. This research shed light on the considerable potential of SLNs and NLCs in delivering antipsychotic drugs directly to the brain via the nasal route, hinting at a new frontier in the treatment of neurological disorders [40,45,47].

In a study by Noorulla et al. [64], chitosan-decorated nanostructured lipid carriers of Buspirone were developed and evaluated for brain targeting. The researchers employed a multifaceted approach, encompassing formulation development, optimization, and in vivo preclinical evaluation. This comprehensive strategy provided valuable insights into the feasibility of utilizing chitosan-decorated nanostructured lipid carriers for enhanced brain targeting and therapeutic efficacy. Tripathi et al. [65] addressed the challenge of brain delivery for Cinnarizine through nanostructured lipid carriers loaded into in situ gel. The research involved thorough in vitro and pharmacokinetic evaluations, establishing the augmented brain delivery of Cinnarizine as a tangible outcome of their innovative formulation strategy. Curcumin-loaded NLCs for nasal administration were investigated by Madane and Mahajan [13]. Their study focused on the design, characterization, and in vivo assessment of the NLCs, providing critical insights into the potential of these lipid-based carriers for nasal drug delivery. Fahmy et al. [66] optimized nanostructured lipid carriers integrated into in situ nasal gel for enhancing brain delivery of Flibanserin. The study not only emphasized the importance of formulation optimization but also highlighted the significance of integrating lipid carriers into a gel matrix for sustained and controlled drug release, ensuring enhanced brain delivery.

Jazuli et al. [67] optimized nanostructured lipid carriers of lurasidone hydrochloride, using the Box–Behnken design, for brain targeting. Their in vitro and in vivo studies underscored the importance of systematic optimization in enhancing the brain-targeting capabilities of lipid-based carriers, emphasizing the potential translational impact of such strategies. The application of computational tools in designing Oleuropein-loaded nanostructured lipid carriers for brain targeting through the nasal route was investigated by Palagati et al. [68]. This study showcased the integration of computational approaches for rational formulation design, highlighting the potential synergy between experimental and computational methodologies in advancing nasal drug delivery strategies.

Wang et al. [69] explored the improved brain delivery of Pueraria flavones via the intranasal administration of borneol-modified solid lipid nanoparticles. Their innovative approach, involving the modification of nanoparticles with borneol, demonstrated enhanced brain delivery, showcasing the versatility of lipid-based carriers in optimizing intranasal drug delivery. Direct brain-targeted nanostructured lipid carriers for the sustained release of a schizophrenic drug were developed by Sivadasu et al. [70]. The study encompassed formulation development, characterization, and pharmacokinetic evaluations, establishing the potential of lipid-based carriers in sustaining drug release and optimizing therapeutic outcomes for psychiatric disorders.

### 4.7. Advantages and Disadvantages of SLNs and NLCs

Solid lipid nanoparticles and NLCs are promising drug delivery systems utilized for nose-to-brain delivery in the treatment of various brain diseases. Solid lipid nanoparticles are composed of a solid lipid core stabilized by surfactants and offer several advantages, including high biocompatibility and biodegradability, which minimize the risk of toxicity and adverse effects. Additionally, SLNs provide controlled release properties, allowing for sustained drug delivery over an extended period. However, SLNs also have limitations, such as a low drug loading capacity and potential drug expulsion during storage, which can compromise their efficacy. One of the most commonly used SLNs in nose-to-brain delivery is the Rivastigmine-loaded SLN developed by Shah et al. [27]. This formulation demonstrated improved drug permeation across the BBB and enhanced therapeutic efficacy in the treatment of Alzheimer’s disease. Despite these advantages, SLNs face challenges related to stability and scalability, limiting their widespread adoption in clinical practice.

In contrast, NLCs overcome some of the drawbacks associated with SLNs by incorporating both solid and liquid lipids, resulting in a more flexible lipid matrix and improved drug loading capacity. This enhanced versatility enables NLCs to accommodate a wider range of hydrophobic and hydrophilic drugs, making them suitable for various therapeutic applications. Additionally, NLCs exhibit superior drug release kinetics and improved tissue penetration compared to SLNs, leading to better therapeutic outcomes. One of the most utilized NLC formulations in nose-to-brain delivery is the astaxanthin-loaded NLC developed by Bhatt et al. [28]. This formulation demonstrated enhanced brain targeting and antioxidant activity, making it a promising candidate for the treatment of neurodegenerative disorders like Alzheimer’s disease. However, NLCs may face challenges related to stability issues, such as lipid crystallization and drug leakage, particularly during long-term storage or exposure to physiological conditions. Furthermore, the complex formulation process and potential batch-to-batch variability associated with NLCs may hinder their widespread adoption in clinical practice.

### 4.8. Challenges and Future Perspectives

While the intranasal administration of lipid nanoparticles, including SLNs and NLCs, has exhibited promising outcomes for enhanced drug delivery to the brain in various studies, several challenges and future perspectives merit consideration. Challenges such as potential toxicity of nanomaterials, precise dosage control, and sustained therapeutic efficacy need to be addressed for the clinical translation of these formulations. Additionally, understanding the intricate interplay between nasal mucosa, the blood–brain barrier, and drug properties is crucial for optimizing delivery strategies. Future research should focus on refining formulation techniques, conducting more extensive pharmacokinetic and pharmacodynamic studies, and exploring the therapeutic potential of these nanoparticles across a spectrum of brain diseases. As we navigate these challenges and delve deeper into the potential of intranasal lipid nanoparticles, a new frontier emerges in the treatment of brain disorders, offering hope for more effective and targeted therapeutic interventions.

## 5. Conclusions

In conclusion, this systematic review sheds light on the revolutionary role of the intranasal administration of lipid nanoparticles in the treatment of brain diseases. The unanimously positive outcomes reported in numerous studies underscore the effectiveness of systems such as SLN and NLC in delivering drugs directly to the brain, overcoming the challenges posed by the blood–brain barrier. This approach holds promising prospects for treating a wide range of cerebral pathologies, from Alzheimer’s disease to stroke. However, as we celebrate these advancements, it is crucial to address remaining challenges, including potential nanoparticle toxicity and the optimization of administration strategies. Through further research and collaborative efforts, the field of intranasal administration of lipid nanoparticles stands poised to drive innovation in brain disease treatment, paving the way for more effective, targeted, and accessible therapies for those in need.

## Figures and Tables

**Table 1:** Summary of the studies included in the systematic literature review reporting on intranasal administration of lipid-based nanocarriers for neuro-oncological lesions.

**Author (Year)**	**Drug Category**	**Drug**	**LN Type**	**Lipid Employed**	**Assays Performed**
Madane et al. (2014) [13]	Phytochemical antiblastic	Curcumin	NLC	Lipids, Precirol, Capmul MCM	Cytotoxicity assay on astrocytoma–glioblastoma cell line U373MG; histopathological studies on sheep nasal mucosa; biodistribution studies on male Wistar rats
Khan et al. (2018) [14]	Antiblastic	Temozolomide	Nanolipid chitosan hydrogel	Gelucire 44/14, vitamin E	Nasal-diffusion study on goat nasal mucosa; brain/plasma uptake studies performed on Wistar rats; cytotoxicity studies on Clone-6 rat glioma cell line
Sousa et al. (2019) [15]	Anti-angiogenic monoclonal antibody	Bevacizumab	PLGA nanoparticles	PLGA	Intranasal bevacizumab efficacy studies in xenograft intracerebral glioblastoma nude mice model
Sintov et al. (2020) [16]	Antiblastic, anti-AD, anti-inflammatory	Curcumin	ALN	Polyoxyl 40 hydrogenated castor oil, CB, tetraglycol, and glyceryl oleate	In vivo administration of curcumin-loaded nanoparticles into Sprague-Dawley rats’ brains
Zhang et al. (2020) [17]	Antiblastic	Paclitaxel	PLGA nanoparticles	PLGA	Cytotoxicity studies on U87MG cells; biodistribution studies in male BALB/c mice
Abd-algaleel et al. (2021) [18]	Antiblastic, antioxidant, anti-inflammatory	Sesamol	SLN, PN	GMS, SA, tristearin, TP, PCL	In vivo pharmacokinetics study on male albino rats
Ahmad et al. (2022) [19]	Antiblastic	Carmustine	Chitosan-coated PLGA nanoparticles	PLGA	Ex vivo permeation study on fresh goat nasal mucosa; pharmacokinetic study in Albino Wistar rats
Sandbhor et al. (2022) [20]	Antiblastic	Paclitaxel and miltefosine	SLN	SPC	Brain retention and biodistribution of intranasal nanoparticles on tumor-free Sprague Daley rats; in vivo anti-glioma efficacy evaluation in orthotopic GBM mice model
Tang et al. (2022) [21]	Nucleic acid drug	siRNA	Nanomicelles	T7-C	Biodistribution of siRNA delivered by T7-C in normal and tumor-bearing mice; creation of an in situ model of GL261 glioma and its therapeutic impact
Trivedi et al. (2023) [22]	Phytochemical with anticancer and antioxidant activity	Thymoquinone	Poly (D-glucosamine) self-assembled lipidic nanovesicles	cholesterol, DSPC	Ex vivo drug permeation study on porcine nasal mucosa of a goat; ex vivo biocompatibility study on goat nasal mucosa; brain uptake study in Albino Wistar rats

Abbreviations:; ALN = amylolipid nanovesicle; BALB/c = albino, laboratory-bred strain of the house mouse; BBB = blood–brain barrier; CB = cocoa butter; DSPC = distearoylphosphatidylcholine; GBM = glioblastoma; GMS = glycerol monostearate; NLC = nanostructured lipid carriers; PA = palmitic acid; PCL = poly cationic lipid; PLGA = poly lactic-co-glycolic acid; PN = polymeric nanoparticle; SA = stearic acid; siRNA = small interfering RNA¸ SLN = solid lipid nanoparticles; SPC = sphingosylphosphorylcholine; T7-C = cholesterol-modified T7; TP = tripalmitin; U87MG = human glioblastoma cell line.

**Table 2:** Summary of the studies included in the systematic literature review reporting on intranasal administration of lipid-based nanocarriers for neurodegenerative disorders.

Author (Year)	Drug Category	Drug	LN Type	Lipid Employed	Assays Performed
Li et al. (2012) [23]	Anti-AD	Galantamine hydrobromide	liposomes	Soya phosphatidylcholine, cholesterol	Rat brain pharmacokinetic behavior, determination cytotoxicity in rat pheochromocytoma PC-12 cell line
Yang et al. (2013) [24]	Anti-AD	Rivastigmine	Liposomes, CPP liposomes	EPC, DSPE-PEG-CPP	Pharmacodynamic study in male Sprague-Dawley rats, evaluation of nasal toxicity
Pardeshi et al. (2013) [2]	Anti-PD	Ropinirole hydrochloride	CASLN	Trimyristin	Ex vivo mucosal toxicity studies on sheep nasal mucosa; anti-tremor activity in albino mice model
Zhao et al. (2013) [25]	Anti-PD	bFGF	GNL	N/A	Pharmacodynamics of intranasal delivery of bFGF-GNLs in hemiparkinsonian rats
Bhatt et al. (2014) [26]	Anti-HD	Rosmarinic acid	SLN	GMS	Functional tests in HD Wistar rat models
Shah et al. (2015) [27]	Anti-AD	Rivastigmine	SLN	Compritol 888 ATO, TPGS	Ex vivo permeation and toxicity studies on sheep nasal mucosa
Chandra Bhatt et al. (2016) [28]	Anti-AD	Astaxanthin	SLN	SA	Biodistribution in male albino Wistar rats
Muntimadugu et al. (2016) [29]	Anti-AD	Tarenflurbil	SLN	Glycerol monostearate, SA, soya lecithin	Biodistribution studies in Sprague-Dawley rats
Rassu et al. (2017) [30]	Anti-AD	BACE1 siRNA	CASLN	Witepsol E 85 solid triglycerides	Permeability studies on Caco-2 cell culture
Esposito et al. (2017) [31]	Anti-MS	Dimethyl fumarate	SLN, CASLN	Tristearin	Biodistribution studies in mice
Yasir et al. (2017) [32]	Anti-AD	Donepezil	SLN	GMS	In vitro release and release kinetic studies; pharmacokinetic and biodistribution in male albino Wistar rats
Gadhave et al. (2019) [5]	Anti-MS	Teriflunomide	NLC	Compritol 888 ATO, Maisine 35–1, Gelucire 44/14	Ex vivo permeation of nanoparticles on nasal mucosa; subacute toxicity evaluation in male Wistar rats
Jojo et al. (2019) [33]	Anti-AD	Pioglitazone	NLC	TP, Capmul MCM	Ex vivo permeation study and nasal ciliotoxicity studies on sheep nasal mucosa; biodistribution study in male Wistar rats
Rajput et al. (2019) [34]	Anti-AD	Resveratrol	NLC	palmitate, Capmul MCM	Pharmacokinetic and biodistribution studies on rats
Gaba et al. (2019) [35]	Anti-PD	Vitamin E	NRG NE	Labrasol, different oils (namely soybean oil, almond oil, olive oil, vitamin E, grape seed oil, rice bran oil, and linseed oil)	In vitro release study, ex vivo permeation study on nasal mucosa; pharmacokinetic and brain-targeting studies in Wistar rats
Jiang et al. (2019) [36]	Anti-AD	Huperzine A	NE, NE modified with lactoferrin	soybean oil, isopropyl myristate, Capryol 90	In vitro studies in hCMEC/D3; test for nasal toxicity in Wistar rats; drug distribution in rat brain
Arora et al. (2020) [37]	Anti-HD	Tetrabenazine	NE	different oil (Capmul MCM, soya bean oil, grape seed oil, and vitamin E)	Ex vivo nasal mucosa permeation study on porcine nasal mucosa; pharmacokinetic and brain delivery study in Wistar rats
Zhang et al. (2020) [38]	Anti-AD	Curcumin	Chitosan-coated poly (lactic-co-glycolic acid) nanoparticles	acetic acid, ethyl acetate	Cytotoxicity and cellular uptake studies in SH-SY5Y cells and BV-2 cells; biodistribution studies in male C57BL/6 mice
Musumeci et al. (2022) [39]	Anti-AD	Anti-TRAIL monoclonal antibody	lipid and polymeric nanocarriers	Cetyl palmitate, glyceryl monooleate, isopropyl stearate	In vivo studies in 3xTg-AD mice and wild type mice: experimental groups and intranasal drug administration

Abbreviations: AD = Alzheimer’s disease; ALS = amyotrophic lateral sclerosis; BACE1 = major -secretase responsible for amyloid-β (Aβ); bFGF = basic fibroblast growth factor; BV-2 = mice microglia cells; CASLN = cationic SLN; CPP = cell penetrating peptide; DSPE-PEG-CPP = 1,2-Distearoyl-sn-glycero-3-phosphoethanolamine-N-[amino (polyethylene glycol)2000]; EPC = egg phosphatidylcholine; GMS = glyceryl monostearate; GNL = gelatin nanostructured lipid carriers; hCMEC/D3 = primary human brain microvascular endothelial cell line; HD = Huntington’s disease; LNS = lipid-based nanosystem; MS = multiple sclerosis; N/A = not applicable; NE = nanoemulsion; NLC = nanostructured lipid carriers; NRG NE = naringenin nanoemulsion; PD = Parkinson’s disease; PGMC = propylene glycol monocaprylate; SA = stearic acid; SH-SY5Y = human neuroblastoma cells; siRNA = small interfering RNA; SLN = solid lipid nanoparticle; TRAIL = TNF related apoptosis-inducing ligand; TP = tripalmitin; TPGS = D-α-tocopherol polyethylene glycol 1000 succinate.

**Table 3:** Summary of the studies included in the systematic literature review reporting on intranasal administration of lipid-based nanocarriers for other brain diseases.

Author (Year)	Drug Category	Drug	LN Type	Lipid Employed	Assays Performed
Patel et al. (2011) [40]	Antipsychotic	Risperidone	SLN	Glyceryl behenate	Biodistribution and paw test in BALB/c mice
Eskandari et al. (2011) [41]	Antiepileptic	Valproic acid	NLC	Cetyl palmitate	Biodistribution and MES seizure test in Wistar rats
Joshi et al. (2012) [42]	Antiemetic	Ondansetron	SLN	GMS	Biodistribution in New Zealand rabbit; histological studies on isolated sheep nasal mucosa
Singh et al. (2012) [43]	Sedative	Alprazolam	SLN	GMS	Biodistribution in New Zealand rabbit
Morsi et al. (2013) [44]	Anti-ischemic	Vinpocetine	SLN bioadhesive	GMS	Ex vivo bioadhesive strength, histopathological, and permeation studies; biodistribution and pharmacokinetics
Gupta et al. (2017) [45]	Antiviral	Efavirenz	SLN	TP, tristearin glyceryl monostearate, glyceryl behenate	Biodistribution in Wistar rats
Fatouh et al. (2017) [46]	Antidepressant	Agomelatine	SLN	TP	Biodistribution in rats
Singh et al. (2017) [47]	Antipsychotic drug	Asenapine maleate	GC-ANLC	GMS, oleic acid	Pharmacokinetic study in Charles Foster rats; embryo fetal toxicity study
Du et al. (2019) [48]	Antifungal drug	Ketoconazole	NLC	Miglyol 812 N, Compritol 888 ATO	In vitro antifungal activity; animal studies in female C57BL/6 mice
Patel et al. (2020) [49]	Antiepileptic	Topiramate	NE	Capmul MCM C8	Pharmacokinetic study and brain drug uptake study in Wistar albino rats
Hosny et al. (2020) [50]	Antiviral	Saquinavir mesylate	Cubosomes	Monoolein	Ex vivo permeation study; in vivo evaluation in albino male rabbits.

Abbreviations: ALS = amyotrophic lateral sclerosis; BALB/c = albino, laboratory-bred strain of the house mouse; C57BL/6 = common inbred strain of laboratory mouse; GC-ANLC = glycol chitosan coated nanostructured lipid carrier; GMS = glyceryl monostearate; LNS = lipid-based nanosystem; NE = nanoemulsion; PGMC = propylene glycol monocaprylate; NLC = nanostructured lipid carriers; SLN = solid lipid nanoparticles; TP = tripalmitin.

## Data Availability

Data available in a publicly accessible repository.
